# Risk of *Mycobacterium tuberculosis* transmission in an antiretroviral therapy clinic

**DOI:** 10.1097/QAD.0000000000002006

**Published:** 2018-10-10

**Authors:** Themba Mzembe, Estelle Mclean, Palwasha Y. Khan, Olivier Koole, Lifted Sichali, Venance Mwinuka, Michael Kayange, Peter Mzumara, Andrew Dimba, Amelia C. Crampin, Judith R. Glynn

**Affiliations:** a Malawi Epidemiology and Intervention Research Unit, Malawi; b Department of Infectious Disease Epidemiology, London School of Hygiene & Tropical Medicine, London, UK; c Ministry of Health; d Karonga District Hospital, Malawi.

**Keywords:** antiretroviral therapy, HIV, infection control, Malawi, risk factors, tuberculosis

## Abstract

**Objective:**

The risk of transmission of *Mycobacterium tuberculosis* in antiretroviral therapy (ART) clinics is recognized, particularly, when HIV and tuberculosis services are unified, but the degree of potential exposure to patients with infectious tuberculosis has not been measured. We aimed to quantify this clinic exposure.

**Methods:**

Over 1 year, we recorded all visits to a clinic in northern Malawi that offers HIV testing and counselling, HIV care, ART, and TB diagnostic and treatment services. We included patients and guardians, noting timing and reason for the visit, using a palm vein reader to assist recognition of individuals and record times automatically. Screening for tuberculosis was enhanced, including induced sputum if necessary.

**Results:**

Information was collected on 5011 individuals and 19 426 visits. During the period, 90 individuals with bacteriologically confirmed pulmonary tuberculosis attended the clinic when they were likely to have been infectious (taken as 6 weeks before diagnosis to 2 weeks after the start of treatment), including 76 who attended before tuberculosis was diagnosed or suspected. We estimated that 19% of visits had at least 1 h of potential exposure to patients with infectious tuberculosis, half to patients attending prediagnosis.

**Conclusion:**

There was considerable risk of exposure, including of immunosuppressed patients, to patients with infectious tuberculosis, especially as repeated visits are made. Much of this exposure could not be avoided by separation of patients with known tuberculosis. Good ventilation and avoidance of crowding is essential to minimize transmission of *M. tuberculosis* in this type of setting.

## Introduction

HIV infection greatly increases the risk of tuberculosis (TB), and even those established on antiretroviral therapy (ART) remain at increased risk of TB compared with people without HIV infection [1]. The proportion of individuals initiating ART who have prevalent TB can be very high [2,3]: for example, 8–18% in South Africa [4–6]. Undiagnosed TB in those attending for ART, pre-ART care, or HIV testing and counselling (HTC) poses a risk of transmission to other clinic attendees. In addition, patients already diagnosed with TB may continue to attend general clinics for HTC or ART, adding to potential exposure.

Countries with dual HIV–TB epidemics are exhorted to integrate HIV and TB services to improve continuity of care [7]. This includes physical integration of facilities, further bringing together people with infectious TB (with or without HIV) and people with immunosuppression.

Although the risk of acquiring TB in HTC/ART clinics is recognized, it has not been quantified. In northern Malawi, we have shown that about 90% of *Mycobacterium tuberculosis* transmission is from unknown contacts [8–10]. *M. tuberculosis* transmission is most likely to occur in poorly ventilated indoor settings [11,12]. ART clinics are settings where transmission from casual contacts is likely. Clinics are held indoors and are frequently crowded, reducing effective ventilation. In an HTC/ART/TB clinic in a district hospital in Malawi, we measured clinic exposure to infectious TB.

## Methods

Between July 2014 and June 2015, we recorded all attendances at the largest HTC/ART/TB clinic in Karonga District, Malawi. The clinic is free-standing with a single entrance, and rooms for HTC, HIV/ART and TB services (including collection and submission of sputum containers for diagnosis, and monitoring, registration, and drug dispensing). There are separate indoor waiting/counselling areas designated for different services, but patients mingle. All patients and their guardians attending the clinic were eligible to participate in the study. At the first visit, the study was explained and written informed consent obtained. Parents/guardians gave consent for children. Identifying information was collected, including using a digital palm vein reader. At all visits, the reason for the visit was collected, and attendees were asked to use the palm vein reader on arrival and departure to aid speedy recognition and automatically record the times of clinic entry and exit.

We have been studying TB in Karonga District since 1986, with enhanced passive surveillance in clinics to improve case finding in those with symptoms. For this study, additional TB screening was carried out for patients initiating ART, and those who had been on ART for 3 and 6 months, with a brief symptom screen [13], and sputum collection (whether or not they were symptomatic), using a nebulizer (outside the clinic building) to induce sputum if necessary. Sputum specimens were processed at the hospital and the project laboratory in Chilumba, Malawi. Sputum that was smear-negative at the hospital was tested by GeneXpert. Positive cultures were sent to the United Kingdom for species confirmation. For those diagnosed with TB, smear and culture results and treatment dates were recorded. The date of diagnosis was taken as the collection date of the first positive specimen, or the start of TB treatment if earlier.

Analyses describe time spent in the clinic by people attending for different reasons. The extent of potential exposure to TB was estimated from the duration of overlap at the clinic with people with TB that was likely to be infectious. This was taken as patients with pulmonary TB that was bacteriologically confirmed [i.e. sputum smear (if more than a single scanty smear) or culture/GeneXpert positive], in the period between 6 weeks before diagnosis to 2 weeks after the start of treatment, or for the entire treatment period if the treatment outcome was ‘failure.’ To adjust for the lower infectiousness of patients with smear-negative pulmonary TB, the time overlap with these patients was divided by 10. As exposure to undiagnosed TB is hardest to avoid, exposure to TB prediagnosis in patients not attending because of suspected TB was examined separately. The analysis was repeated considering only smear-positive TB as infectious.

These estimates are deliberately conservative to estimate minimum exposure: the average time from symptoms to diagnosis is more than 6 weeks [14], and the time from infectiousness onset to treatment will be longer; culture positivity after treatment often persists beyond 2 weeks even with drug-sensitive TB [15,16]; and the proportion of transmission from smear-negative patients may be more than 10% [17–19].

Approval for the study was given by the National Health Sciences Research Committee, Malawi, and the ethics committee of the London School of Hygiene & Tropical Medicine, UK.

## Results

During the study, 5011 individuals attended the clinic at least once, contributing 19 426 visits. No study refusals were noted, but a few people may have bypassed the registration process. Visits for which entry or exit time was missing (*n* = 3690) were taken as the median duration.

### Reasons for attendance

Of the attendees, 3186 (63.6%) were female patients. At their first visit during the study, 250 (5.0%) attendees were aged under 15 years, 3853 (76.9%) aged 15–49 and 906 (18.1%) aged over 50. Three thousand, eight hundred and forty-six (76.8%) attendees were HIV-positive, 427 (8.5%) attended for HTC (but were not found to be HIV positive), 601 (12.0%) attended only as a guardian/caregiver (attending with a patient or to collect medication), and 137 (2.7%) attended for other/unknown reasons. Two hundred and fifty-one (5.0%) attendees were known to be current TB patients and/or gave TB as their reason for attending, including 60 initially attending because of suspected TB. Of 116 patients with bacteriologically confirmed TB, 26 only attended the clinic after being on treatment for at least 2 weeks, but 90 (81 smear-positive, 9 smear-negative) attended the clinic at least once in their ‘infectious’ period, including 76 who attended before TB was diagnosed and who did not give suspected TB as the reason for attendance.

### Time spent in the clinic

The median time spent in the clinic per visit was 81 min [interquartile range (IQR) 42–140], longer for those already known to be HIV-positive (83, IQR 44–144), guardians (72, IQR 42–127) and those attending for HTC (72, IQR 42–107), and shorter for those with bacteriologically confirmed TB (50, IQR 31–92), or suspected TB (48, IQR 17–81).

### Exposure to infectious tuberculosis

**Fig. 1:**
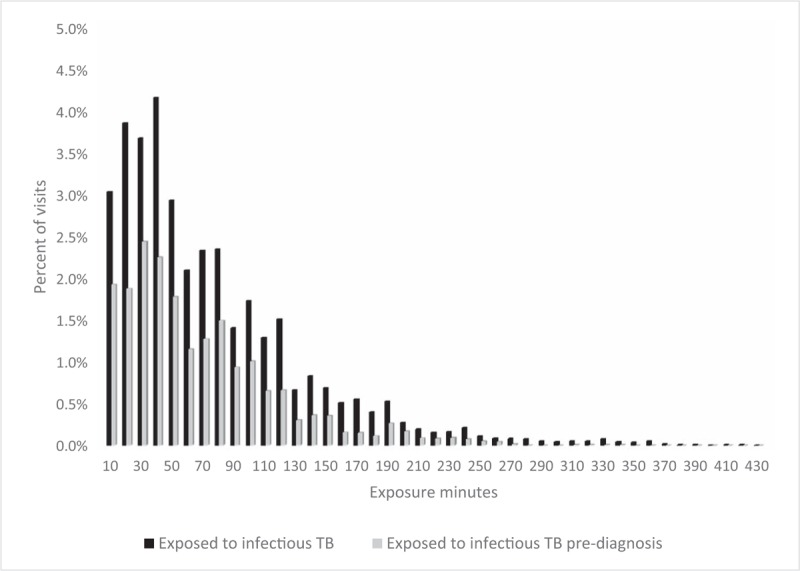
Proportion of visits by minutes exposed to patients with infectious tuberculosis, showing those with at least 10 min exposure.

Overall, there were 18 595 visits by patients not recorded as having had TB: 10 812 (58.1%) of these had no clinic exposure to patients with infectious TB, and 3473 (18.7%) had at least 1 h of potential exposure (Table 1), including 1768 (9.5%) with at least 1 h of potential exposure to patients with undiagnosed infectious TB. The distribution of exposure times is shown in Figure 1. Regarding only smear-positive patients as infectious made little difference to the results: 59.9% of visits had no exposure and 18.6% had at least 1 h exposure (Table, Supplemental Digital Content).

In addition, 64% of visits had less than 10 min exposure to infectious TB and 80% had less than 10 min exposure to infectious TB prediagnosis. TB, tuberculosis.

Potential exposure was lower in children: 23.5% of visits by children aged under 15 vs. 42.9% of visits by adults had some exposure to infectious TB (chi-squared *P* < 0.001); and in visits with exposure, median duration 42 min for children vs. 52 min for adults, (Wilcoxon rank-sum test *P* = 0.002). Visits by men were slightly more likely to have some exposure to infectious TB than those by women (44.4 vs. 40.6%, *P* < 0.001), but in exposed visits, there was little difference between the exposure times (median 50 min for men vs. 52 min for women, *P* = 0.8). Prolonged exposure (over an hour) was more common for those receiving HIV care (17.3%) and on ART (19.2%) than for those receiving only HIV testing (12.0%), or attending as a guardian (15.9%, *P* < 0.001), Table 1.

Many patients made multiple visits during the year of the study (median 4, IQR 2–5, range 1–24). Over the year, 51.8% of attendees had a cumulative exposure to infectious TB of at least 1 h, and 23.0% had at least 3 h exposure. Considering only exposure to undiagnosed infectious TB, 31.4% had a cumulative exposure of at least 1 h and 8.7% had exposure of at least 3 h.

## Discussion

In this study in a busy ART clinic, we estimated that 42% of visits had some potential exposure to patients with infectious TB, and 19% of visits had prolonged exposure (over an hour). Over the year, half the attendees had cumulative potential exposure to infectious TB of an hour or more.

To estimate exposure, we made conservative assumptions about the infectious period. We made even more conservative assumptions in the sensitivity analysis, which only considers smear-positive patients as infectious. Even with these assumptions, there is substantial potential exposure.

In the study, we could only measure overlapping times, not proximity of exposure. The presence of someone with infectious TB in the clinic does not imply high exposure for all patients attending at the same time. But the clinic is often crowded with patients sitting close together on benches or crowding in doorways, and ventilation is limited. Some visits had considerable opportunities for exposure, and as people attend repeatedly, the likelihood of actual exposure is not negligible.

In this setting, children attend the clinic on a different day, which explains their lower exposure, as most people with infectious TB are adults. At the time of the study, HIV-positive individuals initiating treatment were seen at the end of the clinic, potentially increasing exposure, but this policy has changed since test-and-treat started in July 2016.

Exposure to infectious TB could theoretically be reduced by separating those with known or suspected TB from other patients. Such separation is a ‘strong recommendation’ in the WHO guidelines [20]. However, about half of the prolonged exposure was to undiagnosed TB (Table 1), even though diagnostic delay in this setting was minimized by enhanced screening. As some degree of patient mixing is inevitable, the key public health response is to try and reduce transmission, through good ventilation, reducing waiting times and crowding, and ensuring cough etiquette (covering the mouth, moving outside) [20]. In a hot climate, natural ventilation with open waiting areas, high ceilings and open windows on opposite sides can be effective [21]. Simple administrative measures could also help, for example, numbered tickets to prevent people pushing forward to be seen, and to allow them to wait outside if waiting times are prolonged.

There is increasing interest in documenting social mixing patterns to improve understanding of the spread of infections [22]. We have used novel methods to characterize mixing with patients with infectious TB in an HTC/ART clinic, where the presence of immunosuppressed patients makes the consequences of exposure to infection particularly dangerous. The patient mix and experience in this clinic are likely to be typical of many HTC/ART clinics in sub-Saharan Africa. We have quantified the high potential for exposure to infectious TB, much of which was from patients with undiagnosed TB. Good ventilation and systems to avoid crowding are essential to minimize the risk of *M. tuberculosis* transmission in such settings.

## Acknowledgements

Author contributions: The study was conceived by A.C. with J.G., P.K., T.M., M.K. It was conducted by T.M. with P.K., O.K., L.S., V.M., P.M., A.D. It was analyzed by E.M. and J.G. It was written by J.G. with contributions by all other authors.

Source of funding: The study was funded by The Wellcome Trust (grant no. 098610/Z/12/Z).

### Conflicts of interest

There are no conflicts of interest.

## Supplementary Material

Supplemental Digital Content

## Figures and Tables

**Table 1:** Potential clinic exposure to infectious TB by patient characteristics.

	Exposure to infectious TB								Exposure to undiagnosed infectious TB								
	None		<10 min		<1 h		>1 h		None		<10 min		<1 h		>1 h		Total
Overall	10 812	58.1%	1023	5.5%	3286	17.7%	3474	18.7%	14 357	77.2%	559	3.0%	1911	10.3%	1768	9.5%	18 595
Age																	
<15	755	76.5%	25	2.5%	110	11.1%	97	9.8%	841	85.2%	19	1.9%	63	6.4%	64	6.5%	987
15–49	8229	58.4%	803	5.7%	2478	17.6%	2591	18.4%	10 879	77.2%	440	3.1%	1462	10.4%	1320	9.4%	14 101
50+	1821	52.1%	194	5.5%	697	19.9%	786	22.5%	2629	75.2%	99	2.8%	386	11.0%	384	11.0%	3498
Sex																	
Male	3392	55.6%	345	5.7%	1168	19.1%	1197	19.6%	4632	75.9%	185	3.0%	681	11.2%	604	9.9%	6102
Female	7420	59.4%	678	5.4%	2118	17.0%	2277	18.2%	9725	77.8%	374	3.0%	1230	9.8%	1164	9.3%	12 493
Reason for visit																	
HIV+ not on ART	275	57.9%	26	5.5%	92	19.4%	82	17.3%	346	72.8%	12	2.5%	68	14.3%	49	10.3%	475
HIV+ on ART	9158	57.7%	870	5.5%	2800	17.6%	3052	19.2%	12 282	77.3%	481	3.0%	1586	10.0%	1531	9.6%	15 880
HIV+ on ART unknown time	1697	65.9%	109	4.2%	394	15.3%	377	14.6%	2075	80.5%	66	2.6%	222	8.6%	214	8.3%	2577
HIV+ on ART <3 months	557	54.1%	80	7.8%	161	15.6%	231	22.4%	786	76.4%	30	2.9%	101	9.8%	112	10.9%	1029
HIV+ on ART ≥3 months	6904	56.2%	681	5.5%	2245	18.3%	2444	19.9%	9421	76.8%	385	3.1%	1263	10.3%	1205	9.8%	12 274
HIV-testing (not known HIV+)	368	65.0%	36	6.4%	94	16.6%	68	12.0%	429	75.8%	26	4.6%	68	12.0%	43	7.6%	566
Guardians	995	60.7%	91	5.6%	293	17.9%	260	15.9%	1277	77.9%	40	2.4%	183	11.2%	139	8.5%	1639
Unknown	16	45.7%	0	0.0%	7	20.0%	12	34.3%	23	65.7%	0	0.0%	6	17.1%	6	17.1%	35

The number and proportion of visits are shown, with exposure estimated as minutes in the clinic at the same time as patients with infectious TB, overall, and restricted to exposure to undiagnosed TB. TB, tuberculosis.
