# Supplementary material for: Risk of Mycobacterium tuberculosis transmission in an antiretroviral therapy clinic
Source: AIDS. 2018 Oct 10;32(16):2417–21. doi: 10.1097/QAD.0000000000002006 (PMC6200384; doi:10.1097/QAD.0000000000002006)
Supplement: Supplemental Digital Content [file aids-32-2417-s001.doc]

|  | Exposure to infectious TB | | | | | | | |  | Exposure to undiagnosed infectious TB | | | | | | | |  |
| --- | --- | --- | --- | --- | --- | --- | --- | --- | --- | --- | --- | --- | --- | --- | --- | --- | --- | --- |
|  | None | | <10minutes | | <1hour | | >1hour | |  | None | | <10minutes | | <1hour | | >1hour | | Total |
|  |  |  |  |  |  |  |  |  |  |  |  |  |  |  |  |  |  |  |
| **Overall** | 11134 | 59.9% | 704 | 3.8% | 3290 | 17.7% | 3467 | 18.6% |  | 14558 | 78.3% | 360 | 1.9% | 1912 | 10.3% | 1765 | 9.5% | 18595 |
| **Age** |  |  |  |  |  |  |  |  |  |  |  |  |  |  |  |  |  |  |
| <15 | 765 | 77.5% | 15 | 1.5% | 110 | 11.1% | 97 | 9.8% |  | 850 | 86.1% | 10 | 1.0% | 63 | 6.4% | 64 | 6.5% | 987 |
| 15-49 | 8483 | 60.2% | 552 | 3.9% | 2481 | 17.6% | 2585 | 18.3% |  | 11038 | 78.3% | 283 | 2.0% | 1463 | 10.4% | 1317 | 9.3% | 14101 |
| 50+ | 1879 | 53.7% | 136 | 3.9% | 698 | 20.0% | 785 | 22.4% |  | 2662 | 76.1% | 66 | 1.9% | 386 | 11.0% | 384 | 11.0% | 3498 |
| **Sex** |  |  |  |  |  |  |  |  |  |  |  |  |  |  |  |  |  |  |
| Male | 3,503 | 57.4% | 235 | 3.9% | 1,169 | 19.2% | 1,195 | 19.6% |  | 4,699 | 77.0% | 118 | 1.9% | 682 | 11.2% | 603 | 9.9% | 6102 |
| Female | 7,631 | 61.1% | 469 | 3.8% | 2,121 | 17.0% | 2,272 | 18.2% |  | 9,859 | 78.9% | 242 | 1.9% | 1,230 | 9.8% | 1,162 | 9.3% | 12493 |
| **Reason for visit** |  |  |  |  |  |  |  |  |  |  |  |  |  |  |  |  |  |  |
| HIV+ not on ART | 278 | 58.5% | 23 | 4.8% | 92 | 19.4% | 82 | 17.3% |  | 348 | 73.3% | 10 | 2.1% | 68 | 14.3% | 49 | 10.3% | 475 |
| HIV+ on ART | 9453 | 59.5% | 578 | 3.6% | 2802 | 17.6% | 3047 | 19.2% |  | 12462 | 78.5% | 303 | 1.9% | 1586 | 10.0% | 1529 | 9.6% | 15880 |
| HIV+ on ART unknown time | 1743 | 67.6% | 63 | 2.4% | 394 | 15.3% | 377 | 14.6% |  | 2106 | 81.7% | 35 | 1.4% | 222 | 8.6% | 214 | 8.3% | 2577 |
| HIV+ on ART <3 months | 587 | 57.0% | 50 | 4.9% | 162 | 15.7% | 230 | 22.4% |  | 798 | 77.6% | 18 | 1.7% | 102 | 9.9% | 111 | 10.8% | 1029 |
| HIV+ on ART ≥ 3 months | 7123 | 58.0% | 465 | 3.8% | 2246 | 18.3% | 2440 | 19.9% |  | 9558 | 77.9% | 250 | 2.0% | 1262 | 10.3% | 1204 | 9.8% | 12274 |
| HIV-testing (not known HIV+) | 371 | 65.5% | 33 | 5.8% | 94 | 16.6% | 68 | 12.0% |  | 432 | 76.3% | 23 | 4.1% | 68 | 12.0% | 43 | 7.6% | 566 |
| Guardians | 1016 | 62.0% | 70 | 4.3% | 295 | 18.0% | 258 | 15.7% |  | 1293 | 78.9% | 24 | 1.5% | 184 | 11.2% | 138 | 8.4% | 1639 |
| Unknown | 16 | 45.7% | 0 | 0.0% | 7 | 20.0% | 12 | 34.3% |  | 23 | 65.7% | 0 | 0.0% | 6 | 17.1% | 6 | 17.1% | 35 |
|  |  |  |  |  |  |  |  |  |  |  |  |  |  |  |  |  |  |  |

Appendix Table: Exposure to infectious TB by patient characteristics, considering only patients with smear positive TB as infectious. The number of visits is shown, with exposure estimated as minutes in the clinic at the same time as patients with infectious TB, overall, and restricted to exposure to undiagnosed TB.
